# Regionale Unterschiede und Trends in gesunder Lebenserwartung in Deutschland

**DOI:** 10.1007/s00103-024-03864-y

**Published:** 2024-04-12

**Authors:** Elke Loichinger, Thomas Skora, Markus Sauerberg, Pavel Grigoriev

**Affiliations:** 1 Bundesinstitut für Bevölkerungsforschung (BiB), Friedrich-Ebert-Allee 4, 65185 Wiesbaden, Deutschland; 2 GESIS – Leibniz-Institut für Sozialwissenschaften, B6, 4–5, 68159 Mannheim, Deutschland

**Keywords:** Partielle Lebenserwartung, Partielle gesunde Lebenserwartung, Regionale Unterschiede, Gesundheitsbezogene Lebensqualität, Kompression, Partial life expectancy, Partial healthy life expectancy, Regional differences, Health-related quality of life, Compression

## Abstract

**Hintergrund:**

Vor dem Hintergrund steigender Lebenserwartung stellt sich die Frage, in welchem Gesundheitszustand die hinzugewonnenen Lebensjahre verbracht werden. Ziel der vorliegenden Untersuchung ist die erstmalige Berechnung regional differenzierter Unterschiede in gesunder Lebenserwartung für Deutschland.

**Methoden:**

Das Konzept der gesunden Lebenserwartung erlaubt es, regionale Unterschiede in Gesundheitszustand und Sterblichkeit in einer Maßzahl zu vereinen. Im vorliegenden Beitrag kommt das Konzept der partiellen gesunden Lebenserwartung zum Einsatz. Mit amtlichen Daten zu Todesfällen und Bevölkerungszahlen berechnen wir verkürzte Sterbetafeln. Die Daten des Sozio-oekonomischen Panels (SOEP) werden zur Ermittlung der alters- und geschlechtsspezifischen Prävalenzen des Gesundheitszustands herangezogen. Die Analyse regionaler Unterschiede erfolgt anhand einer Einteilung Deutschlands in 4 Regionen (Norden, Süden, Osten, Westen) von 2002 bis 2019.

**Ergebnisse:**

Die regionalen Unterschiede in der gesunden Lebenserwartung in Deutschland sind größer als Unterschiede in der Lebenserwartung an sich und Trends der gesunden Lebenserwartung verlaufen teilweise anders als die entsprechenden Trends der Sterblichkeit. Diese Unterschiede im zeitlichen Verlauf variieren des Weiteren nach Alter: Während es bei der Bevölkerung zwischen 20 und 64 Jahren in der Tendenz zu einer Stagnation, teilweise zu Rückgängen in der gesunden Lebenserwartung gekommen ist, nahmen die Anzahl und der Anteil der Jahre in guter Gesundheit bei den Älteren bis Alter 79 zu.

**Fazit:**

Es gibt auffällige regionale Unterschiede und Trends in der Verteilung der erwarteten Jahre in guter Gesundheit in Deutschland. Die rechtzeitige Identifikation regional abweichender Entwicklungen kann dazu beitragen, gezielte gesundheitsfördernde Maßnahmen zu ergreifen.

**Zusatzmaterial online:**

Zusätzliche Informationen sind in der Online-Version dieses Artikels (10.1007/s00103-024-03864-y) enthalten.

## Abstract (en)

**Background:**

Against the background of increasing life expectancy, the question arises in which state of health the additional years of life are spent. The aim of this study is to assess for the first time regional differences in healthy life expectancy for Germany.

**Methods:**

The concept of healthy life expectancy allows for the combination of regional differences in health status and mortality in a single measure. This article uses the concept of partial healthy life expectancy. We use official data on deaths and population numbers to calculate abridged life tables. Data from the Socio-Economic Panel (SOEP) are used to determine the age- and sex-specific prevalences of health status. Regional differences are analyzed from 2002 to 2019 by dividing Germany into four regions (North, South, East, West).

**Results:**

The regional differences in healthy life expectancy in Germany are greater than differences in life expectancy, and trends in healthy life expectancy partly differ from the corresponding trends in mortality. These differences over time also vary according to age: while healthy life expectancy has tended to stagnate and, in some cases, decline among the population aged between 20 and 64, the number and proportion of years in good health has increased among older adults up to the age of 79.

**Conclusion:**

There are striking regional differences and trends in the distribution of expected years in good health in Germany. The timely identification of regionally divergent developments could facilitate the implementation of targeted health-promoting measures.

## Hintergrund

Die Lebenserwartung bei Geburt ist in Deutschland in den vergangenen Jahrzehnten deutlich gestiegen. Im Jahr 2022 lag sie laut Berechnungen des Statistischen Bundesamtes bei 78,2 Jahren für Männer und 82,9 Jahren für Frauen. Eine in diesem Zusammenhang immer wieder aufkommende Frage ist, in welchem Gesundheitszustand die gewonnenen Lebensjahre verbracht werden. Die Antwort hierauf hat weitreichende Folgen für den Einzelnen wie auch für die Gesellschaft. Maßnahmen der öffentlichen Gesundheit und der Bedarf an Gesundheits- und Pflegeversorgung hängen direkt mit dem Gesundheitszustand der (älteren) Bevölkerung zusammen. Dabei spielt nicht nur die steigende Lebenserwartung eine Rolle, sondern auch die aufgrund der niedrigen Geburtenraten erfolgende Verschiebung in der Altersstruktur, die durch eine anteilige Zunahme der älteren Bevölkerung gekennzeichnet ist. Diese Entwicklung geht mit Fragen der Finanzierbarkeit der Gesundheitsversorgung als auch der personellen Ausstattung im Gesundheits- und Pflegesektor einher.

Die Lebenserwartung ist ein verbreitetes Maß zur Messung der grundsätzlichen Gesundheit einer Bevölkerung. Sie beruht auf Daten zur Sterblichkeit und erlaubt keine weiteren Rückschlüsse darauf, in welchem Gesundheitszustand die Lebensjahre verbracht werden. In Deutschland sind regionale Unterschiede in der Lebenserwartung bekannt und untersucht. Auf Kreisebene betragen sie bei Männern mehr als 5 Jahre, bei Frauen fast 4 Jahre [1]. Diese Unterschiede werden mit sozioökonomischer Deprivation und damit zusammenhängenden Verhaltensweisen in Verbindung gebracht (siehe z. B. [1, 2]) sowie mit Gesundheitsverhalten wie Rauchen [3].

Im Unterschied zur Lebenserwartung gehen in die Berechnung der gesunden Lebenserwartung zusätzlich zu Daten der Sterblichkeit explizite Angaben zum Gesundheitszustand ein. Dadurch werden Aussagen zur Lebensqualität möglich: Wie viele Jahre werden in guter Gesundheit, wie viele in schlechter Gesundheit verbracht? Für die Messung des Gesundheitszustands werden je nach Fragestellung verschiedene Gesundheitsindikatoren herangezogen. Beispielhaft sei die Verwendung der subjektiven Selbsteinschätzung des Gesundheitszustands, das Vorhandensein funktionaler Beeinträchtigungen bei Tätigkeiten des täglichen Lebens oder das Vorliegen von Pflegebedürftigkeit genannt. Wenn es darum geht, Aussagen zur Entwicklung der gesunden Lebensjahre Hochaltriger zu treffen, bietet sich die Betrachtung der Lebensjahre mit Pflegebedarf an (siehe Studie auf regionaler Ebene für Deutschland von Kreft und Doblhammer (2016); [4]). Häufig liegt das Interesse auf der Frage, wie sich die steigende Lebenserwartung im Zeitverlauf auf Jahre in guter oder schlechter Gesundheit verteilt. Bei einem Anstieg der Lebensjahre in guter Gesundheit wird von „Kompression der Morbidität“ gesprochen, bei einer Zunahme in schlechter Gesundheit von „Ausweitung (Expansion) der Morbidität“ (vgl. [5]).

Neben der genannten Studie von Kreft und Doblhammer (2016) gibt es nach den Recherchen der Autorinnen und Autoren keine weiteren Studien, die Berechnungen zur gesunden Lebenserwartung in Deutschland unterhalb der nationalen Ebene beinhalten [4]. Zwar gibt es eine Vielzahl an Studien, die sich mit regionalen Unterschieden im Gesundheitszustand und im Gesundheitsverhalten der Bevölkerung auseinandergesetzt haben [6–9]. Was jedoch weitestgehend fehlt, sind Analysen zu regionalen Unterschieden und Entwicklungen der gesunden Lebenserwartung, sprich, der Kombination aus regionalen Informationen zu Sterblichkeit und Gesundheitszustand. Eine Ausnahme stellt eine aktuelle Studie von Porst et al. (2022) dar, in der auf Ebene der Raumordnungsregionen die Anzahl an Lebensjahren, die aufgrund von Sterblichkeit verloren oder mit gesundheitlicher Einschränkung verbracht werden, berechnet wird [10]. Das dort verwendete Konzept der „Disability-adjusted Life Years“ (DALY) ist neben dem Konzept der gesunden Lebensjahre ein etabliertes gesundheitsbezogenes Summenmaß [11]. Ergebnisse zu Unterschieden in Lebenserwartung, Lebensbedingungen und Gesundheitszustand auf regionaler Ebene sind nicht zuletzt vor dem Grundsatz der Gleichwertigkeit der Lebensverhältnisse relevant. International gesehen ist die gesunde Lebenserwartung ein bewährtes Maß, um gesundheitliche Entwicklungen räumlich und zeitlich zu vergleichen [12–17].

Aufgrund der bekannten regionalen Unterschiede in der Lebenserwartung und im Gesundheitszustand ist davon auszugehen, dass auch in Deutschland regionale Unterschiede in der gesunden Lebenserwartung bestehen. Diese zu berechnen und zu analysieren, ist das Ziel der vorliegenden Studie. Das geschieht unter Verwendung regionaler Informationen der Sterblichkeit und des Gesundheitszustands von 2002 bis 2019 für 4 Regionen, getrennt für Männer und Frauen und für ausgewählte Altersgruppen. Neben der absoluten Anzahl an Jahren in guter Gesundheit betrachten wir, wie sich der Anteil der Lebenserwartung in guter Gesundheit an der Lebenserwartung entwickelt hat. Von Interesse sind dabei sowohl Trends innerhalb von Regionen als auch die Entwicklung von Unterschieden zwischen Regionen und die Frage, ob es unterschiedliche Muster für Männer und Frauen sowie verschiedene Altersgruppen gibt.

## Methoden

**Abb. 1:**
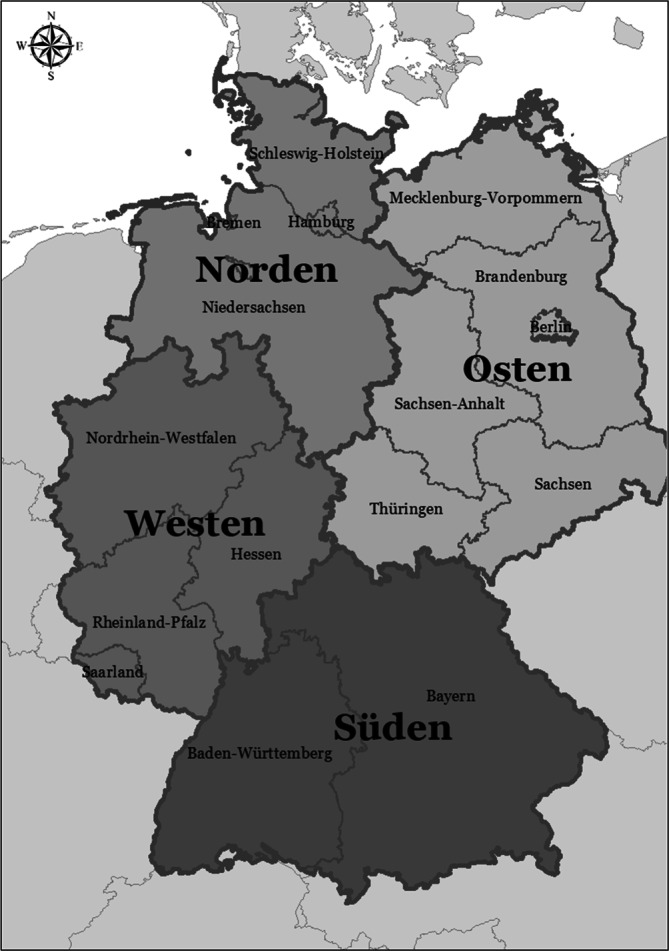
Regionale Gliederung. *Quelle*: Geodaten von www.bkg.bund.de, eigene Darstellung

Zur Berechnung der Lebenserwartung in Gesundheit kombinierten wir amtliche Daten zu Todesfällen und Bevölkerungszahlen sowie Umfragedaten zum Gesundheitszustand der Bevölkerung. Die Analyse regionaler Disparitäten erfolgte anhand einer Einteilung Deutschlands in 4 Regionen, wobei sich die Bundesländer wie folgt verteilten: Norden (Schleswig-Holstein, Hamburg, Niedersachsen, Bremen), Süden (Bayern, Baden-Württemberg), Westen (Nordrhein-Westfalen, Hessen, Rheinland-Pfalz, Saarland) und Osten (Berlin, Brandenburg, Mecklenburg-Vorpommern, Sachsen, Sachsen-Anhalt, Thüringen; Abb. 1).

### Datengrundlage

Amtliche Daten zu Todesfällen und Bevölkerungszahlen beruhen auf den offiziellen Statistiken der Bundesländer. Wir haben die Methoden der Human Mortality Database (HMD) verwendet, um altersspezifische Sterbefälle und die entsprechende Risikobevölkerung für einzelne Kalenderjahre für die 4 Regionen getrennt nach Geschlecht zu erhalten [18].

Die Wahl der Datenquelle zu Gesundheitsinformationen fiel auf das Sozio-oekonomische Panel (SOEP; [19]). Im Vergleich verschiedener potenzieller Datenquellen zeichnete sich das SOEP durch die Länge der verfügbaren Zeitreihe, die vorliegenden Variablen zur Erfassung des Gesundheitszustands, die Stichprobengröße sowie vor allem auch durch die Möglichkeit, räumlich disaggregierte Analysen durchzuführen, aus. Das SOEP wird regelmäßig zur Analyse von gesundheitlichen Fragen herangezogen, zum Beispiel im Rahmen der Gesundheitsberichtserstattung [20]. Die Analysen beruhen auf dem SOEP-Release v37 [21]. Wir verwendeten Daten der Jahre 1996 bis 2019, wobei der Fokus auf dem Zeitraum 2002 bis 2019 lag (siehe auch Fußnote 1).

Die Hauptvariable, die wir zur Berechnung des Gesundheitszustands heranzogen, beruht auf einer leicht modifizierten Version des SF12-Fragebogens (siehe [22]). Dieser wird seit 2002 im 2‑jährigen Rhythmus eingesetzt. Der SF12-Fragebogen ist ein international verwendetes und erprobtes Instrument zur Messung der gesundheitsbezogenen Lebensqualität. Basierend auf 12 Einzelfragen wird im SOEP je eine zusammengefasste Variable zu körperlicher und geistiger Gesundheit erstellt. Klar et al. (2021) verwenden diese beiden Variablen für ihre Berechnungen der gesunden Lebenserwartung für Deutschland (ohne regionale Differenzierung; [23]). Wir konzentrierten uns auf die Variable zu körperlicher Gesundheit (Physical Component Score, PCS), welche u. a. auf Fragen zum gegenwärtigen Gesundheitszustand, zur physischen Gesundheit und zu körperlichen Schmerzen beruht. Im SOEP wurde diese Variable basierend auf den Ausprägungen im SOEP 2004 auf den Wertebereich 0 bis 100 transformiert, ebenso erfolgte eine Standardisierung auf den Mittelwert 50 und eine Standardabweichung von 10. Wir orientierten uns bei der Definition des Gesundheitszustands an Klar et al. (2021) und kategorisierten Personen mit Ausprägungen unter 40 (d. h. einer Standardabweichung unter dem Mittelwert) als stark eingeschränkt bzw. als einen schlechten Gesundheitszustand aufweisend [23].

Zur Überprüfung der Robustheit unserer Ergebnisse zogen wir die Frage zur Selbsteinschätzung des Gesundheitszustands im SOEP heran. Diese Variable wird seit 1994 jährlich erhoben. Die 5 Antwortmöglichkeiten sind „sehr gut“, „gut“, „zufriedenstellend“, „weniger gut“ und „schlecht“. In diesem Fall kategorisierten wir „weniger gut“ und „schlecht“ als schlechten Gesundheitszustand. Ergebnisse für dieses Gesundheitsmaß finden sich im zusätzlichen Onlinematerial.

Die Berechnung der alters- und geschlechtsspezifischen Anteile in schlechter Gesundheit – die sogenannten Prävalenzen – erfolgte für 5‑Jahres-Altersgruppen (20–24, … 75–79). Um stabile Prävalenzprofile zu erhalten war es notwendig, die Daten mehrerer Erhebungswellen zu poolen. Daraus ergaben sich die Analysezeiträume 1996–2001^1^, 2002–2007, 2008–2013 und 2014–2019. Trotz des relativ großen Stichprobenumfangs des SOEP war es dennoch nicht möglich, die Berechnungen der Prävalenzen des Gesundheitszustands nach Alter und Geschlecht kleinräumiger als für die 4 beschriebenen Großregionen durchzuführen. Die verwendete Einteilung in Norden, Süden, Osten und Westen beruht auf der historischen Entwicklung der Wirtschaft und Sterblichkeit in Deutschland [24]. Diese Einteilung deckt sich mit den Regionen, die das Robert Koch-Institut kürzlich in einer Gesundheitserhebung der Bevölkerung 65+ verwendet hat [25].

### Berechnung

Die amtlichen Daten zu Todesfällen und Bevölkerungszahlen flossen in die Berechnung der Lebenserwartung als auch der gesunden Lebenserwartung ein. Das Maß der Lebenserwartung wurde mithilfe von Periodensterbetafeln getrennt nach Periode, Region und Geschlecht berechnet [26]. Die einzelnen Funktionen in einer Sterbetafel basieren auf den altersspezifischen Sterberaten für 5‑Jahres-Altersgruppen (20–24, … 75–79), um sie mit den Gesundheitsdaten aus dem SOEP zu harmonisieren. Die restliche Lebenserwartung im Alter x kann wie folgt ermittelt werden:

1 $$e_{x}=\frac{Tx}{lx}{,}$$

wobei *Tx* die Anzahl der noch zu lebenden Personenjahre im Alter *x* angibt, während *lx* die Anzahl der Überlebenden in der Sterbetafelbevölkerung bis zum Alter *x* darstellt.

Wir können keine verlässlichen Aussagen über den Gesundheitszustand von Personen über 80 Jahren treffen, da im SOEP nur Personen in Privathaushalten und nicht in Gemeinschaftsunterkünften (z. B. in stationärer Pflege) enthalten sind. Daher stellten wir Berechnungen sowohl der Lebenserwartung als auch der gesunden Lebenserwartung nur bis einschließlich Alter 79 an. Um dies methodisch umzusetzen und auch um die Entwicklung der gesunden Lebenserwartung für verschiedene Alter zu analysieren, bietet sich das Konzept der partiellen Lebenserwartung an. Die partielle Lebenserwartung zwischen Alter *x* und *x* *+* *i* gibt an, wie viele Jahre eine Person im Alter x zwischen dem Alter x und x + i durchschnittlich noch lebt. Sie kann folgendermaßen berechnet werden:

2 $${_{i}}e_{x}=\frac{T_{x}-T_{x+i}}{lx}.$$

Wenn wir für x das Alter 20 setzen und i der Zahl 65 entspricht, berechnet Gl. 2 die durchschnittliche Anzahl an Lebensjahren, die eine Person im Alter 20 bis zum 65. Geburtstag erwarten kann. Demensprechend ist die partielle Lebenserwartung ein hilfreicher Indikator für Vergleiche von Sterblichkeitsverhältnissen in bestimmten Altersintervallen [27].

In einer Sterbetafel können auch Informationen zum Gesundheitsstatus berücksichtigt werden, indem die Anzahl der gelebten Personenjahre für die Sterbetafelbevölkerung in Personenjahre in guter und schlechter Gesundheit aufgeteilt wird [28]. Für diese Aufteilung können altersspezifische Prävalenzen verwendet werden,

3 $$_{n}{L}_{x}^{\text{Gesund}}={_{n}}L_{x}\cdot \left(1-{_{n}}\pi _{x}\right){,}$$

wobei ${_{n}}L_{x}$ die Anzahl der gelebten Personenjahre zwischen dem Alter *x* und *x* *+* *n* angibt. Die altersspezifischen Prävalenzen,${_{n}}\pi _{x}$, geben an, wie hoch der Anteil der Personen in schlechter Gesundheit in einer Altersgruppe ist. Diese Informationen basieren in der vorliegenden Studie auf einer Auswertung des SOEP (siehe Abschnitt Datengrundlage).

Die Anzahl der noch zu lebenden Personenjahre in Gesundheit ergibt sich aus der kumulierten Anzahl der gelebten Personenjahre in Gesundheit,

4 $${T}_{x}^{\text{Gesund}}=\overset{\omega}{\underset{a=x}{\sum}}{\ }_{n}{L}_{a}^{\text{Gesund}}.$$

Nun können ähnliche Formeln wie (1) und (2) verwendet werden, um die gesunde Lebenserwartung im Alter *x* sowie die partielle Lebenserwartung in Gesundheit zwischen Alter *x* und *x* *+* *i* zu bestimmen,

5 $${e}_{x}^{\text{Gesund}}=\frac{Tx^{\text{Gesund}}}{lx}{,}$$

6 $${_{i}e}_{x}^{\text{Gesund}}=\frac{{T}_{x}^{\text{Gesund}}-{T}_{x+i}^{\text{Gesund}}}{lx}.$$

Wir berechneten die partielle Lebenserwartung und die partielle Lebenserwartung in Gesundheit für die Bevölkerung im Haupterwerbsalter (20–54 Jahre), für Personen vor und am Anfang des Rentenalters (55–64 Jahre) als auch für Personen im Alter von 65–79 Jahren. Die 95 %-Konfidenzintervalle für die Werte der Lebenserwartung wurden mit der Methode von Chiang (1984) geschätzt [29]. Die Erweiterung dieser Methode von Andreev und Shkolnikov (2010) ermöglicht außerdem die Bestimmung der 95 %-Konfidenzintervalle für die Werte der gesunden Lebenserwartung [30]. Die Breite der 95 %-Konfidenzintervalle für die gesunde Lebenserwartung ergibt sich fast ausschließlich aus der statistischen Unsicherheit in den Gesundheitsdaten (SOEP-Stichprobe), während die regionalen Mortalitätsdaten durch die vergleichsweise großen Fallzahlen (Vollerhebung der amtlichen Statistik) eine deutlich geringe Unsicherheit aufweisen. Berechnungen der Prävalenzen und 95 %-Konfidenzintervalle sowie eine detaillierte Beschreibung von deren Schätzung können Tab. A1 im Onlinematerial entnommen werden.

## Ergebnisse

### Entwicklung der regionalen Lebenserwartung

**Abb. 2:**
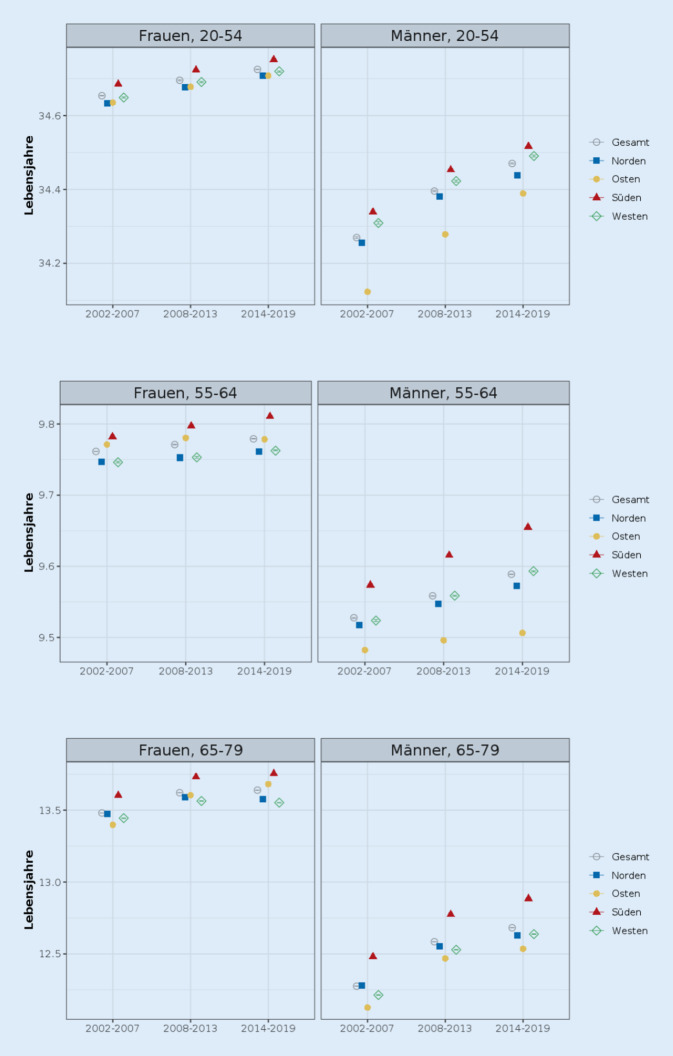
Partielle Lebenserwartung für die Altersgruppen 20–54, 55–64 und 65–79, nach Geschlecht, Zeitraum und Region (in Lebensjahren). *Quelle*: eigene Berechnungen und Abbildung

Die Lebenserwartung ist in allen Regionen und in allen 3 Altersgruppen für Frauen wie Männer im betrachteten Zeitraum angestiegen, wenn auch in unterschiedlichem Ausmaß (Abb. 2 und Tab. A2). Bei den Männern ist die Lebenserwartung durchweg im Süden am höchsten und im Osten am niedrigsten. Bei den Frauen ist sie ebenfalls durchweg im Süden am höchsten, die Region mit den niedrigsten Werten variiert nach Zeitpunkt und Alter. Die Unterschiede in der Lebenserwartung zwischen den Regionen sind bei Frauen deutlich geringer als bei den Männern.

Bei den 20- bis 54-jährigen Männern ging der Unterschied zwischen den Regionen über die Zeit zurück; es fand also eine Konvergenz in der Lebenserwartung statt. Anders bei den 55- bis 64-jährigen Männern: Hier nahm der Unterschied in der Lebenserwartung zwischen den Regionen zu, es kam zu einer Divergenz in dieser Altersspanne. Bei den 65- bis 79-Jährigen gab es keine Veränderung.

Bei den Frauen fallen die Anstiege innerhalb der Regionen über die Zeit deutlich geringer aus als bei den Männern; hier muss jedoch beachtet werden, dass die partielle Lebenserwartung in der jeweiligen Altersgruppe teilweise schon am möglichen Maximalwert liegt – im Alter 55–64 Jahre beträgt dieser 10 Jahre. Für die partielle Lebenserwartung im Alter 20–54 Jahre (bzw. im Alter 65–79 Jahre) liegt er bei 35 Jahren (bzw. 15 Jahren). Die Unterschiede zwischen den Regionen veränderten sich über die Zeit kaum.^2^

### Entwicklung der gesunden Lebenserwartung

Der Anteil der Männer und Frauen mit schlechter Gesundheit nimmt mit steigendem Alter zu. Anteilswerte und die Anzahl der zugrunde liegenden Beobachtungen des SOEP können Tab. A1 im zusätzlichen Onlinematerial entnommen werden.

**Abb. 3:**
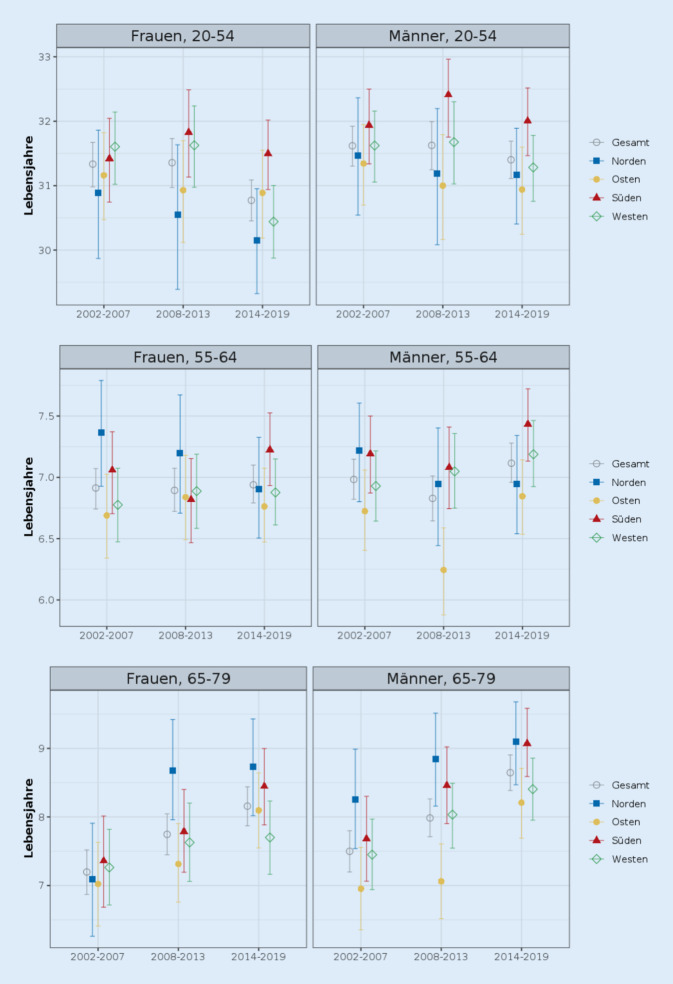
Partielle Lebenserwartung in Gesundheit für die Altersgruppen 20–54, 55–64 und 65–79, nach Geschlecht, Zeitraum und Region (in Lebensjahren; Gesundheitsindikator: körperliche Gesundheit). *Quelle*: eigene Berechnungen und Abbildung

Bei den Männern ist die gesunde Lebenserwartung für die jüngste und mittlere Altersgruppe im aktuellsten Zeitraum (2014–2019) im Süden am höchsten, für die höchste Altersgruppe liegen Süden und Norden nahezu gleichauf (Abb. 3). Die niedrigsten Werte finden sich durchweg im Osten. Bei den Frauen ist sie ebenfalls für die jüngste und mittlere Altersgruppe im aktuellsten Zeitraum im Süden am höchsten, für die höchste Altersgruppe im Norden. Bei der Region mit den niedrigsten Werten ergibt sich bei Frauen kein einheitliches Bild: Norden (Alter 20–54), Osten (Alter 55–64) und Westen (Alter 65–79).

Für Deutschland insgesamt war die Veränderung – ein Anstieg – in der gesunden Lebenserwartung zwischen 2002–2007 und 2014–2019 für 65- bis 79-jährige Männer und Frauen absolut und relativ am größten. Innerhalb der Regionen fanden sich im selben Zeitraum ebenso für die älteste Altersgruppe die größten Veränderungen, wobei es sich gleichfalls um Anstiege handelte (vgl. auch Tab. A3 im Onlinematerial). Parallel dazu zeigen sich bedenkliche Entwicklungen für die jüngere und mittlere Altersgruppe, da hier in der Tendenz die Anzahl gesunder Lebensjahre stagniert oder gesunken ist. Besonders auffällig ist diese Entwicklung für 20- bis 54- und 55- bis 64-jährige Frauen im Norden.

Vergleicht man die Ergebnisse für die Lebenserwartung mit denen der gesunden Lebenserwartung, ist ein Befund, dass sich die Dominanz der südlichen Region bei der Lebenserwartung nicht uneingeschränkt in der gesunden Lebenserwartung widerspiegelt. Zwar führt diese Region im aktuellsten Zeitraum zumeist das Ranking an, in der Vergangenheit war dies jedoch nicht zwangsläufig der Fall. Ein weiterer Befund des Vergleichs beider Maße zeigt, dass die Unterschiede zwischen den Regionen in der gesunden Lebenserwartung größer sind als in der Lebenserwartung an sich. Diese Aussage stimmt für die gesunde Lebenserwartung basierend auf dem Maß für körperliche Gesundheit als auch die subjektive Selbsteinschätzung (siehe Abb. A1 im Onlinematerial) und gilt für Männer wie Frauen aller Altersgruppen und für alle Beobachtungszeitpunkte. Was das Niveau der Lebenserwartung in Gesundheit betrifft, so liegen die Werte für die körperliche Gesundheit der 20- bis 54-jährigen Frauen und Männer über denen der subjektiven Selbsteinschätzung. Bei den 55- bis 64-Jährigen und noch ausgeprägter bei den 65- bis 79-Jährigen ist es umgekehrt und die höheren Werte ergeben sich für die subjektive Selbsteinschätzung.

Was die Konvergenz und Divergenz der Unterschiede zwischen den Regionen betrifft, so zeigen sich bei der gesunden Lebenserwartung andere Muster, als es bei der Lebenserwartung der Fall war: Bei den Männern gab es eine Zunahme der Unterschiede bei den 20- bis 54- und 55- bis 64-Jährigen und einen Rückgang bei den 65- bis 79-Jährigen. Bei den Frauen gab es eine Zunahme der Unterschiede bei den 20- bis 54- und den 65- bis 79-Jährigen sowie einen Rückgang bei den 55- bis 64-Jährigen.

### Entwicklung der Anteile der Lebenserwartung in Gesundheit – Kompression oder Ausweitung?

**Abb. 4:**
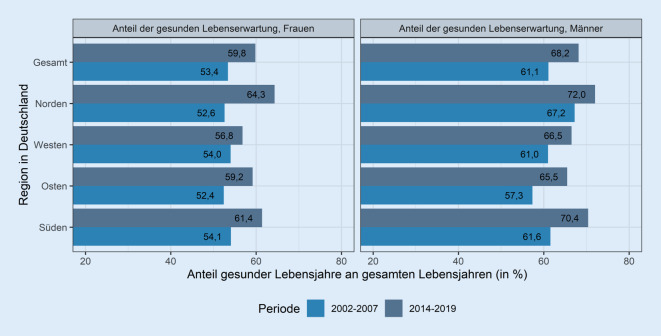
Anteil der partiellen gesunden Lebenserwartung (in Prozent) an der partiellen Lebenserwartung für Altersgruppe 65–79, nach Geschlecht und Region für Zeitraum 2002–2007 und 2014–2019 (Gesundheitsindikator: körperliche Gesundheit). *Quelle*: eigene Berechnungen und Abbildung

Vor dem Hintergrund der Bevölkerungsalterung ist es von großem Interesse, wie sich der Gesundheitszustand gerade der älteren Bevölkerung entwickelt. Abb. 4 zeigt die Entwicklung der Anteile der gesunden Lebenserwartung an der Lebenserwartung bei 65- bis 79-jährigen Frauen und Männern zwischen 2002–2007 und 2014–2019. Hierbei wird deutlich, dass Frauen zwar die höhere Lebenserwartung haben, die Anteile der gesunden Lebenserwartung an der Lebenserwartung jedoch in jeder Region bei den Männern höher sind. Dieses Ergebnis steht im Einklang mit Ergebnissen anderer Studien zu Geschlechtsunterschieden in Mortalität und Morbidität [31, 32]. 2014–2019 lag bei den Männern der Anteil gesunder Lebensjahre mit 65,5 % im Osten am niedrigsten und mit 72,0 % im Norden am höchsten. Bei den Frauen fanden sich die niedrigsten Anteile im Westen (56,8 %) und die höchsten ebenfalls im Norden (64,3 %).

Die Anteile der gesunden Lebenserwartung sind ohne Ausnahme zwischen 2002–2007 und 2014–2019 bei Männern wie Frauen gestiegen: Es kam also zu einer *relativen* Kompression der Morbidität. Da dabei gleichzeitig die absolute Anzahl an Lebensjahren in schlechter Gesundheit gesunken ist (Differenz aus Lebenserwartung und Lebenserwartung in guter Gesundheit), kann man sogar von einer *absoluten* Kompression der Morbidität sprechen (vgl. [23]). Auch dieses Ergebnis gilt für alle Regionen und für Männer wie Frauen ebenso wie für Berechnungen mit dem alternativen Indikator, der auf der subjektiven Selbsteinschätzung des Gesundheitszustands beruht.^3^ Aufgrund einer abweichenden Kategorisierung des selbsteingeschätzten Gesundheitszustands in einer Studie von Sperlich et al. (2019) ist ein direkter Vergleich nur eingeschränkt möglich, jedoch fand sich hier eine relative Kompression der Morbidität für Männer und Frauen für die Altersgruppe 31–90 Jahre, eine absolute Kompression nur bei Frauen bis Alter 60 [33].

Die Unterschiede zwischen den Regionen in den Anteilen gesunder Lebensjahre an den gesamten Lebensjahren haben bei den Frauen zugenommen (d. h. regionale Divergenz, von 2 auf 8 Prozentpunkte) und sind bei den Männern zurückgegangen (d. h. regionale Konvergenz, von 10 auf 7 Prozentpunkte). Gleichzeitig haben sich dadurch die Geschlechterunterschiede zwischen den Regionen verringert.

## Diskussion

Unsere Ergebnisse zeigen, dass die regionalen Unterschiede in der gesunden Lebenserwartung in Deutschland größer sind als Unterschiede in der Lebenserwartung an sich. Darüber hinaus fanden wir, dass Trends bei der gesunden Lebenserwartung teilweise anders verlaufen als Trends in der Sterblichkeit. Diese Unterschiede im zeitlichen Verlauf variieren des Weiteren zwischen den Altersgruppen: Während es bei der Bevölkerung zwischen 20 und 64 Jahren in der Tendenz zu einer Stagnation, teilweise zu Rückgängen in der gesunden Lebenserwartung gekommen ist, nahmen die Anzahl und der Anteil der Jahre in guter Gesundheit bei den Älteren bis Alter 79 zu. Dieser Befund für 4 Regionen Deutschlands deckt sich mit Ergebnissen für Deutschland insgesamt aus früheren Studien zur Entwicklung des Gesundheitszustands in verschiedenen Lebensphasen [23, 33]. Mögliche Erklärungen für die Ergebnisse für Personen im mittleren und späteren Erwerbsalter hängen mit einer Zunahme der physischen und mentalen Belastung in der Arbeitswelt als auch mit dem Einfluss wirtschaftlicher Krisen zusammen [23]. Es besteht hier jedoch weiterer Forschungsbedarf. Ebenso besteht weiterer Analysebedarf, um die in der vorliegenden Studie identifizierte Verschlechterung der Lebensjahre in Gesundheit bei unter 65-jährigen Frauen im Norden einordnen zu können. Nicht zuletzt aufgrund der Vorhersagekraft des Gesundheitszustands für zukünftige Mortalitätstrends ist es wichtig, unterschiedliche Trends zwischen den Altersgruppen frühzeitig zu erkennen und zu verstehen [34, 35]. Ebenso kann die rechtzeitige Identifikation regional abweichender Entwicklungen dazu beitragen, gezielte gesundheitsfördernde Maßnahmen zu ergreifen.

Ziel der vorliegenden Untersuchung war die erstmalige Berechnung regional differenzierter Unterschiede und Trends in gesunder Lebenserwartung für Deutschland. Dabei stellte sich heraus, dass auf Basis verfügbarer Umfragedaten zum Gesundheitszustand der Bevölkerung nur eine großräumige Analyse möglich war. Ursprünglich war eine Analyse auf Bundeslandebene angestrebt worden. Während die Anzahl an Sterbefällen auf Bundeslandebene groß genug ist, um stabile altersspezifische Sterberaten zu berechnen, scheint die SOEP-Stichprobe nicht groß genug zu sein, um selbst bei einer Zusammenlegung mehrerer Erhebungswellen verlässliche Trends in der altersspezifischen Prävalenz in schlechter Gesundheit für einzelne Bundesländer zu erhalten. Starke Fluktuationen zwischen den Altersgruppen sowie über die Zeit ließen keine zuverlässigen Vergleiche zwischen Bundesländern zu. Es wäre zwar möglich gewesen unter Verwendung von statistischen Schätzmodellen die Prävalenzprofile zu glätten. Wir haben uns jedoch aufgrund der dabei in Kauf zu nehmenden Verluste an Transparenz und regionalspezifischen Entwicklungen dagegen entschieden. Stattdessen haben wir unsere Daten in 4 Großregionen aggregiert, was die Berechnung der alters- und geschlechtsspezifischen Prävalenzen in schlechter Gesundheit direkt als Anteilswerte aus den beobachteten Daten ermöglichte. Die visuelle Überprüfung ergab kontinuierliche Verläufe über das Alter und keine ungewöhnlichen Sprünge über die Zeit.

Des Weiteren haben wir uns gegen die Verwendung des sogenannten Multi-State-Modells als Alternative zur Methode nach Sullivan für die Berechnung der gesunden Lebenserwartung entschieden, weil diese Methode Übergangsraten zwischen den einzelnen Gesundheitsstatus benötigt. Diese sind in der Regel unstabiler als Prävalenzen, sodass Regressionsmodelle mit zusätzlichen Annahmen über das erwartete Altersprofil benötigt werden, um Übergangsraten aus Umfragedaten zu ermitteln. Demzufolge ist die statistische Unsicherheit in den Schätzungen der gesunden Lebenserwartung auf Basis der Multi-State-Methode oftmals höher als bei Schätzwerten, die mithilfe der Sullivan-Methode und der beobachteten Prävalenzdaten berechnet werden [36].

Wie bei jeder Berechnung der gesunden Lebenserwartung besteht ein Zusammenhang zwischen der Definition dessen, was gute und schlechte Gesundheit bedeutet, und dem Ergebnis der erwarteten Jahre in Gesundheit. Die Entscheidung für die Verwendung der Variablen zur körperlichen Gesundheit im SOEP hatte mehrere Vorteile. Zum einen handelt es sich hierbei um ein international eingesetztes und erprobtes Instrument zur Messung der gesundheitsbezogenen Lebensqualität. Des Weiteren basiert der damit geschätzte Gesundheitszustand nicht auf einer Einzelfrage, sondern er ist das Ergebnis mehrerer zusammengefasster Variablen, welche seit 2002 unverändert im SOEP erfasst werden. Weitere Umfragen, die regelmäßig in Deutschland durchgeführt werden, erlaubten entweder keine regionale Differenzierung, die verfügbaren Zeitreihen waren deutlich kürzer oder nicht aktuell oder die enthaltenen Variablen zum Gesundheitszustand boten sich nicht für Berechnungen der Lebenserwartung in Gesundheit an.

Im Unterschied zur Sterblichkeit, welche klar definiert und eindeutig messbar ist, gibt es viele Dimensionen von Gesundheit, was es erschwert, den Gesundheitszustand zu bewerten und zu vergleichen. Wir haben versucht, dieser Tatsache durch die Verwendung eines zusätzlichen alternativen Gesundheitsmaßes Rechnung zu tragen. Wenn es im generellen Niveau der Jahre in Gesundheit und in einzelnen Regionen auch Unterschiede zwischen unserem Hauptmaß und dem alternativen Maß gab, so zeigt sich doch, dass sich die grundsätzlichen Trends in den Ergebnissen beider Maße finden.

Da in der Stichprobe des SOEP nur private Haushalte enthalten sind, lässt sich der Gesundheitszustand der Hochaltrigen nicht repräsentativ abbilden: Ab Alter 80 nimmt der Anteil der Bevölkerung in stationärer Pflege generell stark zu und darüber hinaus auch die regionalen Unterschiede der Bevölkerungsanteile in stationärer Pflege. Aus diesem Grund limitierten wir unsere Analysen auf Berechnungen zur partiellen Lebenserwartung und partiellen Lebenserwartung in Gesundheit bis einschließlich Alter 79. Vor dem Hintergrund weiterer Bevölkerungsalterung sind repräsentative Daten der Bevölkerung 80+, die eine regionale Auswertung zulassen, anzustreben. Diesbezüglich sollte die Möglichkeit regionaler Analysen mit einem neuen Datensatz (Studie „Hohes Alter in Deutschland“ (D80+)) geprüft werden [37].

## Fazit

Unsere Ergebnisse zeigen, dass es auffällige regionale Unterschiede und Trends in der Verteilung der erwarteten Jahre in guter Gesundheit in Deutschland gibt. Derartige Entwicklungen bleiben beim reinen Vergleich von Unterschieden und Trends in der Lebenserwartung unentdeckt und zeigen, wie wichtig es ist, regionale Unterschiede im Gesundheitszustand in den Blick zu nehmen. Gerade Anzeichen für stagnierende oder rückläufige Entwicklungen bei der Anzahl der Jahre in guter Gesundheit bei Personen im Haupterwerbsalter können ein Warnsignal sein, nicht nur, was den Gesundheitszustand dieser Bevölkerungsgruppen momentan anbelangt, sondern auch, was dies für den weiteren Lebensverlauf dieser Gruppen bedeutet.

## Supplementary Information

Weitere Tabellen und Abbildungen zu Prävalenzen, Lebenserwartung und Lebenserwartung in Gesundheit

## Footnotes

1 Die Berechnung der gesunden Lebenserwartung ist für diesen Zeitraum nur für den Gesundheitsindikator „Selbsteinschätzung des Gesundheitszustands“ möglich (Ergebnisse siehe zusätzliches Onlinematerial).

2 Die den gezeigten Abbildungen zugrunde liegenden Werte sowie die Ergebnisse für den Gesundheitsindikator „Selbsteinschätzung des Gesundheitszustands“ finden sich im zusätzlichen Onlinematerial.

3 Die Werte für die junge und mittlere Altersgruppe sind in Tab. A4a im zusätzlichen Onlinematerial enthalten. Ergebnisse für subjektive Selbsteinschätzung des Gesundheitszustands finden sich in Tab. A4b und Abb. A2.

## References

[1] Rau R, Schmertmann CP (2020) Lebenserwartung auf Kreisebene in Deutschland. Dtsch Ärztebl Int 117:493–499. 10.3238/arztebl.2020.0493 33087229 10.3238/arztebl.2020.0493 PMC7588608

[2] Kroll LE, Schumann M, Hoebel J, Lampert T (2017) Regionale Unterschiede in der Gesundheit – Entwicklung eines sozioökonomischen Deprivationsindex für Deutschland. J Health Monit 2:103–120. 10.17886/RKI-GBE-2017-035.237168133

[3] Grigoriev P, Klüsener S, van Raalte A (2022) Quantifying the contribution of smoking to regional mortality disparities in Germany: a cross-sectional study. BMJ Open 12:e64249. 10.1136/bmjopen-2022-064249 36180117 10.1136/bmjopen-2022-064249 PMC9528608

[4] Kreft D, Doblhammer G (2016) Expansion or compression of long-term care in Germany between 2001 and 2009? A small-area decomposition study based on administrative health data. Popul Health Metr 14:1–15. 10.1186/s12963-016-0093-1 27418881 10.1186/s12963-016-0093-1 PMC4944474

[5] Unger R (2016) Lebenserwartung in Gesundheit. In: Niephaus Y, Kreyenfeld M, Sackmann R (Hrsg) Handbuch Bevölkerungssoziologie. Springer Fachmedien Wiesbaden, Wiesbaden, S 565–594

[6] Atzendorf J, Apfelbacher C, de Matos EG et al (2020) Do smoking, nutrition, alcohol use, and physical activity vary between regions in Germany?—results of a cross-sectional study. BMC Public Health 20:1–8. 10.1186/s12889-020-8352-2 32164635 10.1186/s12889-020-8352-2 PMC7068923

[7] Diederichs C, Neuhauser H, Kroll L et al (2017) Regionale Unterschiede in der Prävalenz von kardiovaskulären Risikofaktoren bei Männern und Frauen in Deutschland. Bundesgesundheitsbl 60:151–162. 10.1007/s00103-016-2493-6 10.1007/s00103-016-2493-6 28004144

[8] Finger JD, Mensink GB, Lange C, Manz K (2017) Gesundheitsfördernde körperliche Aktivität in der Freizeit bei Erwachsenen in Deutschland. J Health Monit 2:37–44. 10.17886/RKI-GBE-2017-02737377532

[9] Lampert T, Müters S, Kuntz B, Dahm S, Nowossadeck E (2019) 30 Jahre nach dem Fall der Mauer: Regionale Unterschiede in der Gesundheit der Bevölkerung Deutschlands. J Health Monit 4:2–25. 10.25646/607635586335

[10] Porst M, von der Lippe Leddin EJ et al (2022) Krankheitslast in Deutschland und seinen Regionen. Ergebnisse zu den „disability-adjusted life years“ (DALY) aus der Studie BURDEN 2020. Dtsch Artzebl 119(46):785–792 10.3238/arztebl.m2022.0314 PMC9902892 36350160

[11] Murray CJ, Salomon JA, Mathers CD, Lopez AD (Hrsg) (2002) Summary measures of population health: concepts, ethics, measurement and applications. Geneva

[12] Chang M‑H, Molla MT, Truman BI, Athar H, Moonesinghe R, Yoon PW (2015) Differences in healthy life expectancy for the US population by sex, race/ethnicity and geographic region: 2008. J Public Health 37:470–479. 10.1093/pubmed/fdu059 10.1093/pubmed/fdu059 25174043

[13] Liu J, Chen G, Chi I et al (2010) Regional variations in and correlates of disability-free life expectancy among older adults in China. BMC Public Health 10:1–8. 10.1186/1471-2458-10-446 20670431 10.1186/1471-2458-10-446 PMC2920279

[14] Szwarcwald CL, de Souza Júnior PRB, Marques AP, Da Almeida WS, Montilla DER (2016) Inequalities in healthy life expectancy by Brazilian geographic regions: findings from the National Health Survey, 2013. Int J Equity Health 15:1–9. 10.1186/s12939-016-0432-7 27852270 10.1186/s12939-016-0432-7 PMC5112675

[15] Groenewegen PP, Westert GP, Boshuizen HC (2003) Regional differences in healthy life expectancy in The Netherlands. Public Health 117:424–429. 10.1016/S0033-3506(03)00100-8 14522158 10.1016/S0033-3506(03)00100-8

[16] van Oyen H, Tafforeau J, Roelands M (1996) Regional inequities in health expectancy in Belgium. Soc Sci Med 43:1673–1678. 10.1016/s0277-9536(96)00080-9 8961411 10.1016/s0277-9536(96)00080-9

[17] Zheng X‑Y, Xu X‑J, Liu Y‑Y et al (2020) Age-standardized mortality, disability-adjusted life-years and healthy life expectancy in different cultural regions of Guangdong, China: a population-based study of 2005–2015. BMC Public Health 20:1–20. 10.1186/s12889-020-8420-7 32503557 10.1186/s12889-020-8420-7 PMC7275520

[18] Max Planck Institute for Demographic Research (Germany), University of California, Berkeley (USA), French Institute for Demographic Studies (2022) HMD. Human mortality database. www.mortality.org. Zugegriffen: 22. Juni 2022

[19] Goebel J, Grabka MM, Liebig S et al (2019) The German Socio-Economic Panel (SOEP). Jahrb Natl Okon Stat 239:345–360. 10.1515/jbnst-2018-0022

[20] Lampert T, Hoebel J, Kuntz B, Müters S, Kroll LE (2017) Gesundheitliche Ungleichheit in verschiedenen Lebensphasen. Gesundheitsberichterstattung des Bundes. Gemeinsam getragen von RKI und Destatis, Berlin

[21] Sozio-oekonomisches Panel (2022) Version 37, Daten der Jahre 1984–2020 (SOEP-Core, v37, EU Edition)

[22] Nübling M, Andersen HH, Mühlbacher A (2006) Data Documentation 16. Entwicklung eines Verfahrens zur Berechnung der körperlichen und psychischen Summenskalen auf Basis der SOEP – Version des SF 12 (Algorithmus). Berlin

[23] Klar MK, Geyer S, Safieddine B, Tetzlaff F, Tetzlaff J, Sperlich S (2021) Trends in healthy life expectancy between 2002 and 2018 in Germany—compression or expansion of health-related quality of life (HRQOL)? SSM Popul Health 13:100758. 10.1016/j.ssmph.2021.100758 33732863 10.1016/j.ssmph.2021.100758 PMC7937823

[24] Klüsener S, Zagheni E (2014) The east-west gradient in spatial population development within Germany. Hist Methods 47:167–179. 10.1080/01615440.2014.955234

[25] Fuchs J, Gärtner B, Perlitz H et al (2023) Studie zur Gesundheit älterer Menschen in Deutschland (Gesundheit 65+): Zielsetzung, Konzeption und Durchführung. J Health Monit 8:66–90. 10.25646/11662

[26] Preston S, Heuveline P, Guillot M (2003) Demography: measuring and modeling population processes. John Wiley & Sons

[27] Arriaga EE (1984) Measuring and explaining the change in life expectancies. Demography 21:83–96. 10.2307/2061029 6714492

[28] Sullivan DF (1971) A single index of mortality and morbidity. HSMHA Health Rep 86(4):347–354 5554262 PMC1937122

[29] Chiang CL (1984) The life table and its applications. Robert E. Krieger Publishing, Malabar, Florida

[30] Andreev EM, Shkolnikov VM (2010) Spreadsheet for calculation of confidence limits for any life table or healthy-life table quantity, S 2010–2005

[31] Di Lego V, Di Giulio P, Luy M (2020) Gender differences in healthy and unhealthy life expectancy. In: Jagger C, Crimmins EM, Saito Y, de Carvalho Yokota RT, van Oyen H, Robine J‑M (Hrsg) International handbook of health expectancies, Bd. 9. Springer International Publishing, Cham, S S 151–S 172

[32] Idler EL (2003) Discussion: gender differences in self-rated health, in mortality, and in the relationship between the two. Gerontologist 43:372–375. 10.1093/geront/43.3.372

[33] Sperlich S, Tetzlaff J, Geyer S (2019) Trends in good self-rated health in Germany between 1995 and 2014: do age and gender matter? Int J Public Health 64:921–933. 10.1007/s00038-019-01235-y 30918976 10.1007/s00038-019-01235-y

[34] Jylhä M (2011) Self-rated health and subjective survival probabilities as predictors of mortality. In: Rogers RG, Crimmins EM (Hrsg) International handbook of adult mortality, Bd. 2. Springer Netherlands, Dordrecht, S S 329–S 344

[35] Himes CL (2011) Relationships among health behaviors, health, and mortality. In: Rogers RG, Crimmins EM (Hrsg) International handbook of adult mortality, Bd. 2. Springer Netherlands, Dordrecht, S S 289–S 310

[36] Saito Y, Robine J‑M, Crimmins EM (2014) The methods and materials of health expectancy. Stat J IAOS 30:209–223. 10.3233/SJI-140840 30319718 10.3233/SJI-140840 PMC6178833

[37] Kaspar R, Simonson J, Tesch-Römer C, Wagner M, Zank S (2023) Hohes Alter in Deutschland Bd. 8. Springer Berlin Heidelberg, Berlin, Heidelberg

