# Supplementary material for: Regional differences and trends in healthy life expectancy in Germany
Source: Bundesgesundheitsblatt Gesundheitsforschung Gesundheitsschutz. 2024 Apr 12;67(5):546–54. [Article in German] doi: 10.1007/s00103-024-03864-y (PMC11636762; doi:10.1007/s00103-024-03864-y)
Supplement: Supplementary file 1 — Weitere Tabellen und Abbildungen zu Prävalenzen, Lebenserwartung und Lebenserwartung in Gesundheit [file 103_2024_3864_MOESM1_ESM.pdf]

Onlinematerial zum Beitrag „Regionale Unterschiede und Trends in gesunder Lebenserwartung in Deutschland“

Elke Loichinger, Thomas Skora, Markus Sauerberg, Pavel Grigoriev (2024)

Tab. A1: Anteil der Bevölkerung in schlechter Gesundheit nach Geschlecht, Altersgruppe, Zeitraum und Region

Altersgruppe	Zeitraum	Deutschland Gesamt				Norden				Osten				Westen				Süden			
		N	Prävalenz, %			N	Prävalenz, %			N	Prävalenz, %			N	Prävalenz, %			N	Prävalenz, %		
			Est.	low_CI	up_CI		Est.	low_CI	up_CI		Est.	low_CI	up_CI		Est.	low_CI	up_CI		Est.	low_CI	up_CI
**Männer**																					
**20-24**	2002-2007	2201	2,2	1,6	2,9	297	3,3	1,3	5,1	606	2,4	1,3	3,6	717	2,4	1,4	3,6	581	1,3	0,5	2,4
	2008-2013	1764	2,8	2,1	3,6	240	1,7	0,4	3,3	511	3,0	1,6	4,5	533	2,8	1,5	4,3	480	3,3	1,9	5,2
	2014-2019	3567	2,8	2,2	3,3	683	3,2	2,0	4,7	579	3,1	1,7	4,7	1354	2,6	1,8	3,5	951	2,4	1,5	3,5
**25-29**	2002-2007	1939	2,5	1,9	3,2	261	2,9	1,1	5,0	581	3,0	1,7	4,5	555	2,4	1,3	3,6	542	1,8	0,7	3,1
	2008-2013	1560	4,1	3,3	5,1	215	1,4	0,0	3,3	421	7,6	5,0	10,2	492	4,0	2,2	5,7	432	2,3	1,2	3,9
	2014-2019	3248	4,9	4,2	5,7	576	3,9	2,4	5,6	656	4,9	3,4	6,6	1178	5,3	4,1	6,6	838	5,1	3,6	6,7
**30-34**	2002-2007	2492	4,2	3,5	5,0	342	4,1	2,0	6,1	590	4,6	3,1	6,3	861	3,8	2,7	5,2	699	4,4	2,9	5,9
	2008-2013	1561	4,0	3,1	5,0	238	9,1	5,5	13,0	454	4,3	2,6	6,4	434	4,4	2,8	6,2	435	0,5	0,0	1,4
	2014-2019	3534	5,3	4,7	6,0	567	4,1	2,6	5,8	773	6,2	4,7	8,0	1259	6,2	5,0	7,5	935	4,2	2,9	5,5
**35-39**	2002-2007	3249	6,2	5,5	7,1	484	8,8	6,4	11,2	674	5,9	4,3	7,6	1138	5,7	4,5	7,1	953	5,8	4,2	7,3
	2008-2013	1919	4,8	3,9	5,8	258	7,9	4,7	11,2	501	5,2	3,4	7,4	642	4,5	3,0	6,1	518	3,2	1,7	4,8
	2014-2019	3833	6,8	6,0	7,6	610	7,5	5,4	9,7	892	6,6	5,2	8,1	1258	8,4	6,9	10,0	1073	4,8	3,5	6,2
**40-44**	2002-2007	3320	8,0	7,2	8,9	400	7,6	5,0	10,3	831	9,9	8,1	11,9	1147	7,8	6,4	9,4	942	6,9	5,3	8,5
	2008-2013	2527	11,2	10,0	12,4	388	15,5	11,9	19,1	551	12,6	9,8	15,4	859	9,7	7,8	11,5	729	9,6	7,4	11,8
	2014-2019	4102	9,8	8,9	10,7	662	8,6	6,3	10,7	851	12,0	10,0	14,3	1405	10,9	9,3	12,7	1184	7,5	6,0	9,0
**45-49**	2002-2007	3026	12,2	11,0	13,4	365	12,9	9,3	16,4	851	12,1	10,0	14,3	1042	13,2	10,9	15,3	768	10,7	8,6	12,8
	2008-2013	2605	11,8	10,6	13,0	298	12,7	9,4	16,8	671	12,7	10,1	15,4	896	10,7	8,8	12,7	740	12,0	9,9	14,5
	2014-2019	4379	15,3	14,2	16,3	722	18,2	15,6	21,2	868	18,2	15,7	20,7	1562	14,7	13,0	16,6	1227	12,3	10,5	14,1

Altersgruppe	Zeitraum	Deutschland Gesamt				Norden				Osten				Westen				Süden			
		N	Prävalenz, %			N	Prävalenz, %			N	Prävalenz, %			N	Prävalenz, %			N	Prävalenz, %		
			Est.	low_CI	up_CI		Est.	low_CI	up_CI		Est.	low_CI	up_CI		Est.	low_CI	up_CI		Est.	low_CI	up_CI
**50-54**	2002-2007	2759	19,5	18,1	20,9	374	18,1	14,2	22,5	725	20,1	17,2	22,9	942	20,2	17,6	22,8	718	18,8	16,0	21,7
	2008-2013	2485	18,2	16,5	19,8	329	17,4	13,4	21,3	703	22,4	19,2	25,5	830	20,4	17,7	23,0	623	10,9	8,7	13,5
	2014-2019	3868	17,9	16,7	19,1	570	21,7	18,1	24,9	808	19,8	17,1	22,5	1440	17,5	15,6	19,2	1050	15,0	13,0	17,2
**55-59**	2002-2007	2474	23,4	21,7	25,1	374	24,4	20,1	28,9	621	23,4	20,0	26,6	861	23,7	20,7	26,7	618	22,5	19,4	25,7
	2008-2013	2287	27,3	25,4	29,3	305	27,8	23,0	33,1	649	31,0	27,6	34,5	758	26,9	23,6	29,9	575	23,5	20,2	27,0
	2014-2019	3006	21,8	20,3	23,4	423	21,1	17,3	25,1	747	22,5	19,7	25,7	1067	23,5	20,9	26,1	769	19,2	16,6	22,1
**60-64**	2002-2007	2690	30,2	28,5	31,9	412	24,0	20,1	28,2	737	35,2	31,5	38,7	892	31,0	28,0	34,0	649	27,4	24,0	30,8
	2008-2013	2166	29,9	28,0	31,8	314	26,6	22,0	31,8	561	37,7	33,9	41,9	722	25,6	22,4	28,8	569	29,4	25,8	32,9
	2014-2019	2548	30,0	28,1	31,7	391	34,1	29,7	38,6	744	33,7	30,4	37,1	806	26,7	23,6	29,7	607	27,0	23,6	30,3
**65-69**	2002-2007	2293	32,4	30,5	34,1	316	25,9	21,2	31,0	673	36,2	32,5	40,0	788	31,5	28,3	34,8	516	32,7	28,7	36,6
	2008-2013	2254	31,8	29,9	33,8	362	24,3	19,9	28,7	631	38,3	34,4	42,2	694	32,2	28,8	35,7	567	28,9	25,0	32,6
	2014-2019	2264	28,7	26,9	30,6	367	28,7	24,3	33,2	601	27,7	24,1	31,3	711	27,5	24,2	30,7	585	31,4	27,7	35,0
**70-74**	2002-2007	1532	41,9	39,4	44,5	229	33,8	27,5	39,7	413	46,1	41,2	51,1	518	42,3	38,2	46,5	372	41,7	36,8	46,8
	2008-2013	2069	35,5	33,4	37,4	302	30,6	25,5	35,4	614	43,3	39,4	47,4	660	35,7	32,3	39,1	493	28,5	24,5	32,5
	2014-2019	1987	30,4	28,3	32,5	353	25,3	21,2	30,0	504	36,4	32,3	40,7	602	33,1	29,7	36,9	528	24,9	21,2	28,4
**75-79**	2002-2007	923	44,5	41,3	47,7	159	41,2	34,0	47,8	203	47,7	40,9	54,2	312	45,7	39,7	51,0	249	42,4	36,1	48,6
	2008-2013	1187	44,2	41,4	47,0	168	35,4	28,6	42,9	330	50,4	44,8	55,8	393	41,1	36,9	46,1	296	46,4	40,9	52,0
	2014-2019	1724	37,6	35,4	39,8	262	30,2	24,8	35,9	456	41,4	37,1	46,1	555	42,0	37,8	45,8	451	32,7	28,2	36,8
**Frauen**																					
**20-24**	2002-2007	2315	2,7	2,0	3,4	321	2,9	1,2	4,7	682	3,1	1,8	4,4	703	3,1	1,8	4,4	609	1,8	0,8	2,8
	2008-2013	1790	2,7	2,0	3,5	224	4,3	1,8	7,1	482	3,3	1,9	5,0	581	1,9	0,9	3,1	503	2,3	1,2	3,8
	2014-2019	3146	5,2	4,4	6,0	616	5,0	3,4	6,7	490	4,3	2,7	6,1	1172	7,2	5,7	8,7	868	3,3	2,2	4,5
**25-29**	2002-2007	2246	3,8	3,1	4,6	306	5,2	2,9	7,8	582	3,0	1,7	4,5	703	3,2	2,0	4,6	655	4,6	3,1	6,3

Altersgruppe	Zeitraum	Deutschland Gesamt				Norden				Osten				Westen				Süden			
		N	Prävalenz, %			N	Prävalenz, %			N	Prävalenz, %			N	Prävalenz, %			N	Prävalenz, %		
			Est.	low_CI	up_CI		Est.	low_CI	up_CI		Est.	low_CI	up_CI		Est.	low_CI	up_CI		Est.	low_CI	up_CI
	2008-2013	1848	2,8	2,1	3,6	296	4,3	2,0	6,8	494	2,8	1,4	4,3	533	2,9	1,7	4,5	525	1,9	0,8	3,2
	2014-2019	3248	5,6	4,8	6,4	527	7,1	4,9	9,1	758	4,3	3,0	5,8	1146	7,6	6,1	9,2	817	3,0	1,8	4,0
**30-34**	2002-2007	2741	5,3	4,4	6,2	368	7,0	4,3	9,8	585	4,6	3,1	6,3	971	5,3	4,0	6,8	817	4,9	3,5	6,6
	2008-2013	1882	6,0	5,0	7,1	269	8,6	5,6	11,9	515	7,4	5,2	9,7	580	5,7	3,8	7,6	518	3,7	2,1	5,4
	2014-2019	4230	8,6	7,7	9,4	742	9,7	7,7	12,1	944	10,1	8,2	12,2	1440	9,6	8,1	11,0	1104	5,2	3,9	6,6
**35-39**	2002-2007	3441	7,9	7,0	8,8	497	8,6	6,2	10,9	728	6,5	4,5	8,2	1234	7,9	6,4	9,4	982	8,7	6,9	10,4
	2008-2013	2195	6,9	5,8	7,9	294	5,1	2,7	7,8	487	7,4	5,1	9,9	781	6,8	5,2	8,5	633	7,2	5,2	9,3
	2014-2019	4927	8,9	8,1	9,6	762	12,7	10,4	15,0	1055	5,9	4,6	7,4	1679	11,6	10,0	13,0	1431	5,8	4,7	7,0
**40-44**	2002-2007	3586	11,9	10,9	13,1	460	17,0	13,7	20,7	917	12,6	10,5	14,8	1258	9,4	7,9	11,0	951	12,1	10,2	14,2
	2008-2013	2769	11,0	9,8	12,1	410	11,8	8,8	15,1	597	12,7	10,0	15,4	1001	11,1	9,4	13,2	761	9,0	7,0	11,0
	2014-2019	4864	13,8	12,8	14,7	774	13,6	11,1	15,9	960	11,7	9,6	13,8	1711	14,7	13,2	16,4	1419	14,2	12,4	16,3
**45-49**	2002-2007	3338	15,6	14,3	16,7	443	16,3	12,9	19,9	911	17,9	15,6	20,5	1122	12,7	11,0	14,7	862	16,4	14,2	18,9
	2008-2013	2950	16,2	15,0	17,6	387	23,5	19,1	27,6	782	18,1	15,5	20,8	1031	12,1	10,2	14,2	750	16,2	13,6	18,7
	2014-2019	5015	17,2	16,2	18,3	898	18,6	16,1	21,3	964	17,2	14,7	19,5	1783	16,9	15,2	18,6	1370	16,8	15,0	18,8
**50-54**	2002-2007	2924	20,2	18,9	21,7	389	19,1	15,2	22,9	783	23,0	20,3	25,8	1044	20,2	17,9	22,8	708	17,7	15,0	20,6
	2008-2013	2864	22,2	20,5	23,7	377	26,2	22,0	30,8	805	24,5	21,5	27,3	926	21,6	18,8	24,3	756	18,4	15,6	21,2
	2014-2019	4204	20,8	19,6	22,0	655	25,6	22,6	28,9	932	23,9	21,4	26,7	1503	19,0	17,0	20,9	1114	17,7	15,4	19,7
**55-59**	2002-2007	2368	27,0	25,4	28,8	338	24,3	19,8	29,0	614	30,6	27,0	34,5	773	27,1	24,1	30,1	643	24,9	21,8	28,3
	2008-2013	2604	27,3	25,5	28,9	338	27,5	22,8	32,3	748	29,0	25,8	32,4	879	27,8	25,0	30,7	639	24,2	21,0	27,5
	2014-2019	3335	26,3	24,7	27,7	473	26,5	22,4	30,4	885	27,7	24,6	30,4	1058	27,0	24,2	29,5	919	24,0	21,2	26,8
**60-64**	2002-2007	2648	31,4	29,8	33,1	388	24,6	20,4	28,9	729	32,5	29,2	35,7	868	34,0	30,9	37,1	663	30,9	27,6	34,7
	2008-2013	2236	31,7	29,7	33,5	312	24,8	19,9	30,1	613	31,2	27,4	34,9	717	31,0	27,6	34,3	594	36,7	33,2	40,6
	2014-2019	2864	31,9	30,2	33,4	410	32,1	27,6	36,3	826	34,1	30,8	37,3	908	32,2	29,4	35,1	720	28,8	25,4	31,9

Altersgruppe	Zeitraum	Deutschland Gesamt				Norden				Osten				Westen				Süden			
		N	Prävalenz, %			N	Prävalenz, %			N	Prävalenz, %			N	Prävalenz, %			N	Prävalenz, %		
			Est.	low_CI	up_CI		Est.	low_CI	up_CI		Est.	low_CI	up_CI		Est.	low_CI	up_CI		Est.	low_CI	up_CI
**65-69**	2002-2007	2350	39,1	37,1	41,2	340	35,7	30,6	40,9	708	41,2	37,6	44,9	756	40,0	36,5	43,4	546	37,2	32,8	41,6
	2008-2013	2293	35,0	33,1	36,8	377	29,6	24,9	34,2	639	37,7	33,8	41,2	674	36,2	32,5	39,9	603	34,3	30,5	37,8
	2014-2019	2345	33,8	31,8	35,7	371	28,6	24,5	33,4	630	34,2	30,6	37,8	733	35,1	31,5	38,6	611	34,9	31,4	38,5
**70-74**	2002-2007	1542	46,7	44,2	49,2	218	51,8	45,4	58,3	426	48,0	43,2	52,8	524	46,6	42,2	50,8	374	42,4	37,7	47,6
	2008-2013	2143	44,0	41,9	46,1	334	32,6	27,5	37,4	620	48,2	44,2	51,9	664	44,4	40,8	48,5	525	45,7	41,5	49,9
	2014-2019	1980	39,2	37,3	41,4	341	38,9	34,0	44,0	522	38,2	34,1	42,3	573	43,8	39,8	48,0	544	35,6	31,6	39,5
**75-79**	2002-2007	1240	55,6	52,9	58,4	189	56,6	49,7	63,5	312	54,9	49,7	60,3	440	52,5	47,9	57,3	299	60,1	54,8	65,6
	2008-2013	1232	51,8	49,1	54,5	195	48,0	41,0	54,4	347	54,3	49,0	59,4	387	52,0	46,5	56,8	303	51,1	45,5	56,4
	2014-2019	1736	48,8	46,5	51,0	233	40,4	33,9	46,4	513	51,4	47,2	55,8	535	52,1	47,9	56,3	455	46,2	41,5	51,0

Quelle: SOEP, eigene Berechnungen

Gesundheitsindikator: körperliche Gesundheit

N = Anzahl der Beobachtungen, Est.=Anteil der Personen in schlechter Gesundheit (Prävalenz), low_CI=untere Grenze des 95 %-Konfidenzintervalls, up_CI=obere Grenze des 95 %-Konfidenzintervalls

Die Verfahren von Chiang (1984) und Andreev und Shkolnikov (2010) beruhen auf der Annahme, dass die altersspezifischen Sterbefälle oder die Anzahl der Personen mit Gesundheitseinschränkungen statistische Zufallsvariablen sind, die der Binomialverteilung folgen, was die Durchführung von Monte-Carlo-Simulationen ermöglicht (siehe [29] und [30] im Haupttext für eine Ausführliche Beschreibung der Methode). Für unsere Analyse haben wir 1000 Simulationen durchgeführt. Für jede simulierte Anzahl an Sterbefällen oder Personen mit Gesundheitseinschränkungen errechnete sich eine andere Sterbetafel mit einem anderen Wert für die (gesunde) Lebenserwartung. Wir erhielten demnach 1000 verschiedene Schätzungen für die (gesunde) Lebenserwartung. Wenn wir diese Schätzungen der Größe nach sortieren (vom höchsten bis zum niedrigsten Wert), entsprechen die 95 %-Konfidenzintervalle den Rängen 25 und 975. Oder anders gesagt: Die 95 %-Konfidenzintervalle ergaben sich aus den 2,5 und 97,5 Perzentilen der Verteilung für die simulierten Werte der (gesunden) Lebenserwartung.

Tab. A2: Partielle Lebenserwartung (in Jahren) nach Geschlecht, Zeitraum, Altersgruppe und Region

Altersgruppe	Zeitraum	Gesamt	Norden	Osten	Süden	Westen	Diff. zwischen Region mit höchster und niedrigster LE (in Jahren)
**Männer**							
**20-54**	2002-2007	34,27	34,26	34,12	34,34	34,31	0,22
	2008-2013	34,40	34,38	34,28	34,45	34,42	0,17
	2014-2019	34,47	34,44	34,39	34,52	34,49	0,13
**55-64**	2002-2007	9,53	9,52	9,48	9,57	9,52	0,09
	2008-2013	9,56	9,55	9,50	9,62	9,56	0,12
	2014-2019	9,59	9,57	9,51	9,65	9,59	0,15
**65-79**	2002-2007	12,28	12,28	12,13	12,48	12,21	0,35
	2008-2013	12,58	12,55	12,47	12,77	12,53	0,31
	2014-2019	12,68	12,63	12,54	12,89	12,64	0,35
**20-79**	2002-2007	53,33	53,25	52,60	53,91	53,36	1,31
	2008-2013	54,05	53,94	53,35	54,64	54,08	1,29
	2014-2019	54,46	54,27	53,75	55,07	54,49	1,32
**Frauen**							
**20-54**	2002-2007	34,65	34,63	34,64	34,69	34,65	0,05
	2008-2013	34,70	34,68	34,68	34,72	34,69	0,05
	2014-2019	34,73	34,71	34,71	34,75	34,72	0,04
**55-64**	2002-2007	9,76	9,75	9,77	9,78	9,75	0,04
	2008-2013	9,77	9,75	9,78	9,80	9,75	0,04
	2014-2019	9,78	9,76	9,78	9,81	9,76	0,05
**65-79**	2002-2007	13,48	13,48	13,40	13,60	13,44	0,20
	2008-2013	13,62	13,59	13,60	13,73	13,56	0,17
	2014-2019	13,64	13,58	13,68	13,75	13,55	0,20
**20-79**	2002-2007	56,37	56,24	56,29	56,68	56,23	0,45
	2008-2013	56,66	56,50	56,63	56,97	56,50	0,47
	2014-2019	56,81	56,62	56,80	57,14	56,63	0,52

Quelle: SOEP, offizielle Daten aus der amtlichen Statistik, eigene Berechnungen

Tab. A3: Partielle Lebenserwartung in Gesundheit (in Jahren) für die Altersgruppen 20-54, 55-64 und 65-79, nach Geschlecht, Zeitraum und Region, für beide Gesundheitsindikatoren

a) Gesundheitsindikator: körperliche Gesundheit (Physical Component Score, PCS)

Altersgruppe	Zeitraum	Gesamt			Norden			Osten			Süden			Westen		
		HLE	low_CI	up_CI	HLE	low_CI	up_CI	HLE	low_CI	up_CI	HLE	low_CI	up_CI	HLE	low_CI	up_CI
**Männer**																
**20-54**	2002-2007	31,62	31,30	31,92	31,47	30,54	32,36	31,34	30,70	31,95	31,94	31,34	32,50	31,62	31,06	32,16
	2008-2013	31,63	31,25	31,99	31,18	30,08	32,20	31,00	30,16	31,79	32,41	31,75	32,96	31,68	31,03	32,30
	2014-2019	31,40	31,11	31,69	31,17	30,41	31,89	30,94	30,25	31,60	32,00	31,47	32,52	31,28	30,76	31,78
**55-64**	2002-2007	6,98	6,82	7,15	7,22	6,80	7,61	6,72	6,40	7,06	7,19	6,87	7,50	6,93	6,64	7,22
	2008-2013	6,83	6,65	7,01	6,95	6,44	7,40	6,24	5,88	6,59	7,08	6,74	7,41	7,05	6,75	7,36
	2014-2019	7,12	6,96	7,28	6,95	6,54	7,34	6,85	6,54	7,14	7,43	7,13	7,72	7,19	6,92	7,46
**65-79**	2002-2007	7,50	7,20	7,80	8,25	7,54	8,99	6,95	6,35	7,56	7,68	7,06	8,30	7,45	6,94	7,97
	2008-2013	7,99	7,71	8,26	8,84	8,16	9,51	7,06	6,52	7,61	8,46	7,90	9,02	8,03	7,54	8,49
	2014-2019	8,65	8,39	8,90	9,10	8,47	9,68	8,21	7,69	8,71	9,07	8,59	9,58	8,41	7,95	8,86
**Frauen**																
**20-54**	2002-2007	31,33	30,98	31,67	30,89	29,87	31,86	31,16	30,47	31,82	31,42	30,75	32,05	31,60	31,02	32,14
	2008-2013	31,36	30,97	31,73	30,54	29,39	31,63	30,93	30,12	31,70	31,83	31,13	32,49	31,63	30,98	32,24
	2014-2019	30,77	30,45	31,09	30,15	29,32	30,95	30,89	30,19	31,55	31,50	30,94	32,02	30,44	29,88	31,00
**55-64**	2002-2007	6,91	6,74	7,07	7,36	6,93	7,79	6,69	6,34	7,02	7,06	6,70	7,37	6,78	6,47	7,07
	2008-2013	6,89	6,72	7,07	7,20	6,71	7,67	6,84	6,49	7,18	6,82	6,47	7,15	6,89	6,58	7,19
	2014-2019	6,94	6,79	7,10	6,90	6,51	7,33	6,76	6,47	7,07	7,23	6,93	7,53	6,88	6,61	7,15
**65-79**	2002-2007	7,20	6,87	7,52	7,09	6,26	7,91	7,02	6,41	7,63	7,36	6,68	8,01	7,26	6,72	7,82
	2008-2013	7,75	7,45	8,05	8,67	7,96	9,42	7,31	6,76	7,90	7,79	7,19	8,40	7,63	7,06	8,20
	2014-2019	8,16	7,87	8,44	8,73	8,02	9,43	8,10	7,55	8,64	8,45	7,88	9,00	7,70	7,16	8,23

Quelle: SOEP, offizielle Daten aus der amtlichen Statistik, eigene Berechnungen

HLE= gesunde Lebenserwartung in Jahren, low_CI=untere Grenze des 95 %-Konfidenzintervalls, up_CI=obere Grenze des 95 %-Konfidenzintervalls

b) Alternativer Gesundheitsindikator: Selbsteinschätzung des Gesundheitszustands

Altersgruppe	Zeitraum	Gesamt			Norden			Osten			Süden			Westen		
		HLE	low_CI	up_CI	HLE	low_CI	up_CI	HLE	low_CI	up_CI	HLE	low_CI	up_CI	HLE	low_CI	up_CI
**Männer**																
**20-54**	1996-2001	30,79	30,49	31,07	30,65	29,80	31,46	30,55	29,97	31,09	30,73	30,14	31,30	31,05	30,56	31,53
	2002-2007	30,45	30,17	30,72	30,63	29,86	31,37	30,13	29,55	30,68	30,70	30,16	31,22	30,43	29,95	30,90
	2008-2013	30,67	30,40	30,93	30,44	29,71	31,13	30,34	29,78	30,88	31,07	30,58	31,54	30,69	30,23	31,14
	2014-2019	30,52	30,28	30,77	30,45	29,82	31,06	30,15	29,59	30,69	31,10	30,64	31,53	30,30	29,89	30,72
**55-64**	1996-2001	7,04	6,91	7,17	6,96	6,58	7,34	7,16	6,92	7,39	7,07	6,81	7,32	6,94	6,70	7,19
	2002-2007	7,12	7,01	7,24	7,37	7,10	7,66	6,86	6,63	7,10	7,21	6,98	7,43	7,14	6,94	7,33
	2008-2013	7,11	6,99	7,23	7,38	7,09	7,68	6,89	6,64	7,11	7,17	6,94	7,40	7,12	6,91	7,31
	2014-2019	7,28	7,18	7,38	7,19	6,90	7,46	7,12	6,92	7,31	7,58	7,37	7,78	7,23	7,05	7,41
**65-79**	1996-2001	8,44	8,18	8,70	8,96	8,28	9,61	8,22	7,69	8,73	8,58	8,09	9,07	8,22	7,77	8,66
	2002-2007	8,57	8,37	8,77	9,09	8,62	9,55	8,13	7,73	8,51	9,13	8,73	9,52	8,28	7,93	8,62
	2008-2013	9,06	8,87	9,23	9,60	9,15	10,02	8,36	8,02	8,72	9,51	9,16	9,85	9,06	8,74	9,37
	2014-2019	9,80	9,64	9,96	9,81	9,40	10,20	9,45	9,14	9,77	10,11	9,79	10,42	9,80	9,50	10,11
**Frauen**																
**20-54**	1996-2001	30,36	30,04	30,67	30,32	29,41	31,18	30,28	29,69	30,88	30,31	29,71	30,94	30,49	29,94	31,03
	2002-2007	30,19	29,90	30,48	30,03	29,22	30,81	30,09	29,52	30,66	30,38	29,85	30,90	30,21	29,72	30,71
	2008-2013	30,12	29,85	30,37	29,91	29,20	30,58	29,69	29,14	30,23	30,61	30,13	31,09	30,09	29,61	30,55
	2014-2019	29,74	29,48	30,00	29,24	28,60	29,88	29,74	29,14	30,30	30,55	30,09	31,01	29,34	28,88	29,78
**55-64**	1996-2001	7,10	6,97	7,23	7,26	6,88	7,63	6,97	6,72	7,21	7,10	6,81	7,38	7,14	6,90	7,38
	2002-2007	7,34	7,23	7,46	7,82	7,55	8,10	7,21	6,97	7,45	7,24	7,00	7,46	7,31	7,11	7,51
	2008-2013	7,23	7,12	7,35	7,53	7,23	7,81	7,38	7,16	7,59	7,01	6,78	7,24	7,17	6,96	7,37
	2014-2019	7,29	7,18	7,39	7,24	6,96	7,51	7,09	6,90	7,30	7,54	7,36	7,75	7,25	7,06	7,42
**65-79**	1996-2001	8,33	8,07	8,58	8,22	7,52	8,90	8,62	8,16	9,09	8,18	7,61	8,71	8,21	7,77	8,66
	2002-2007	8,50	8,27	8,71	8,40	7,84	8,94	8,47	8,06	8,87	8,78	8,32	9,22	8,40	8,03	8,76
	2008-2013	9,20	8,99	9,39	9,70	9,24	10,17	9,07	8,68	9,46	9,40	9,00	9,79	8,90	8,53	9,25
	2014-2019	9,49	9,30	9,68	9,78	9,32	10,24	9,53	9,17	9,89	9,72	9,36	10,09	9,14	8,79	9,49

Quelle: SOEP, offizielle Daten aus der amtlichen Statistik, eigene Berechnungen

HLE= gesunde Lebenserwartung in Jahren, low_CI=untere Grenze des 95 %-Konfidenzintervalls, up_CI=obere Grenze des 95 %-Konfidenzintervalls

Tab. A4: Anteil der partiellen gesunden Lebenserwartung an der partiellen Lebenserwartung (%) für die Altersgruppen 20-54, 55-64 und 65-79, nach Geschlecht, Zeitraum und Region, für beide Gesundheitsindikatoren

a) Gesundheitsindikator: körperliche Gesundheit (Physical Component Score, PCS)

Altersgruppe	Zeitraum	Gesamt	Norden	Osten	Süden	Westen
**Männer**						
**20-54**	2002-2007	92,3	91,9	91,9	93,0	92,2
	2008-2013	91,9	90,7	90,4	94,1	92,0
	2014-2019	91,1	90,5	90,0	92,7	90,7
**55-64**	2002-2007	73,3	75,8	70,9	75,1	72,8
	2008-2013	71,4	72,8	65,8	73,6	73,7
	2014-2019	74,2	72,6	72,0	77,0	74,9
**65-79**	2002-2007	61,1	67,2	57,3	61,6	61,0
	2008-2013	63,5	70,5	56,6	66,2	64,1
	2014-2019	68,2	72,0	65,5	70,4	66,5
**Frauen**						
**20-54**	2002-2007	90,4	89,2	90,0	90,6	91,2
	2008-2013	90,4	88,1	89,2	91,7	91,2
	2014-2019	88,6	86,9	89,0	90,6	87,7
**55-64**	2002-2007	70,8	75,6	68,5	72,2	69,5
	2008-2013	70,6	73,8	69,9	69,6	70,6
	2014-2019	71,0	70,7	69,2	73,7	70,4
**65-79**	2002-2007	53,4	52,6	52,4	54,1	54,0
	2008-2013	56,9	63,8	53,8	56,7	56,2
	2014-2019	59,8	64,3	59,2	61,4	56,8

Quelle: SOEP, offizielle Daten aus der amtlichen Statistik, eigene Berechnungen

b) Alternativer Gesundheitsindikator: Selbsteinschätzung des Gesundheitszustands

Altersgruppe	Zeitraum	Gesamt	Norden	Osten	Süden	Westen
**Männer**						
**20-54**	1996-2001	90,3	89,9	90,1	89,9	90,8
	2002-2007	88,9	89,4	88,3	89,4	88,7
	2008-2013	89,2	88,5	88,5	90,2	89,1
	2014-2019	88,5	88,4	87,7	90,1	87,9
**55-64**	1996-2001	74,4	73,5	76,1	74,4	73,3
	2002-2007	74,7	77,5	72,4	75,3	74,9
	2008-2013	74,4	77,3	72,5	74,6	74,5
	2014-2019	75,9	75,1	74,9	78,5	75,4
**65-79**	1996-2001	71,4	75,5	70,8	71,4	69,9
	2002-2007	69,8	74,0	67,1	73,1	67,8
	2008-2013	72,0	76,5	67,0	74,4	72,3
	2014-2019	77,3	77,7	75,4	78,4	77,6
**Frauen**						
**20-54**	1996-2001	87,8	87,7	87,6	87,5	88,2
	2002-2007	87,1	86,7	86,9	87,6	87,2
	2008-2013	86,8	86,2	85,6	88,2	86,7
	2014-2019	85,7	84,2	85,7	87,9	84,5
**55-64**	1996-2001	72,8	74,6	71,6	72,8	73,3
	2002-2007	75,2	80,2	73,8	74,0	75,0
	2008-2013	74,0	77,2	75,5	71,6	73,5
	2014-2019	74,5	74,2	72,5	76,9	74,2
**65-79**	1996-2001	63,1	62,1	66,1	61,3	62,2
	2002-2007	63,1	62,3	63,2	64,5	62,4
	2008-2013	67,5	71,4	66,6	68,4	65,7
	2014-2019	69,6	72,1	69,7	70,7	67,5

Quelle: SOEP, offizielle Daten aus der amtlichen Statistik, eigene Berechnungen

Abbildung A1: Partielle Lebenserwartung in Gesundheit für die Altersgruppen 20-54, 55-64 und 65-79, nach Geschlecht, Zeitraum und Region (in Jahren) (Gesundheitsindikator: Selbsteinschätzung des Gesundheitszustands)

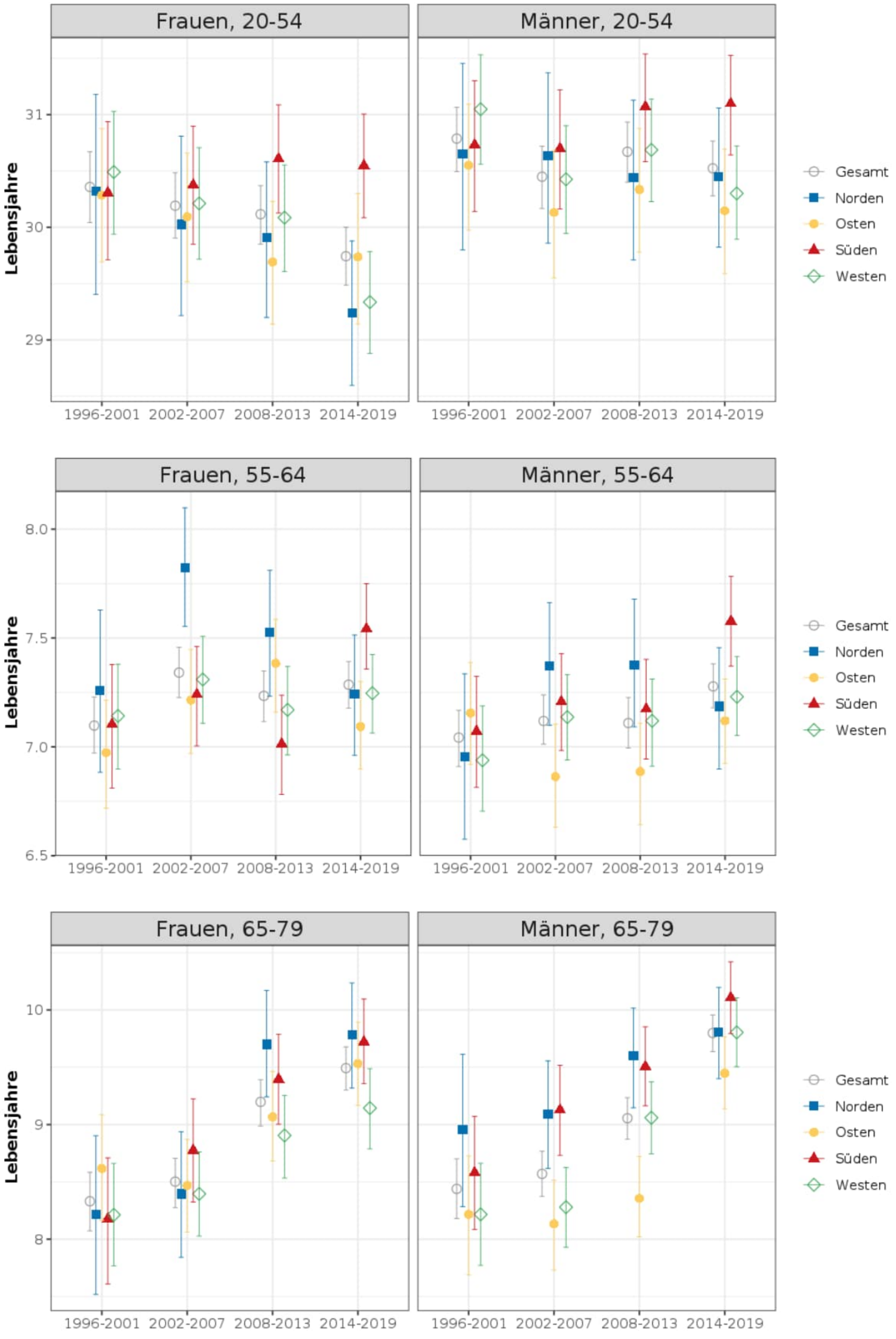

Quelle: Eigene Berechnungen und Abbildung

Abbildung A2: Anteil der partiellen gesunden Lebenserwartung (in Prozent) an der partiellen Lebenserwartung für die Altersgruppe 65-79, nach Geschlecht und Region für die Zeiträume 2002-2007 und 2014-2019 (Gesundheitsindikator: Selbsteinschätzung des Gesundheitszustands)

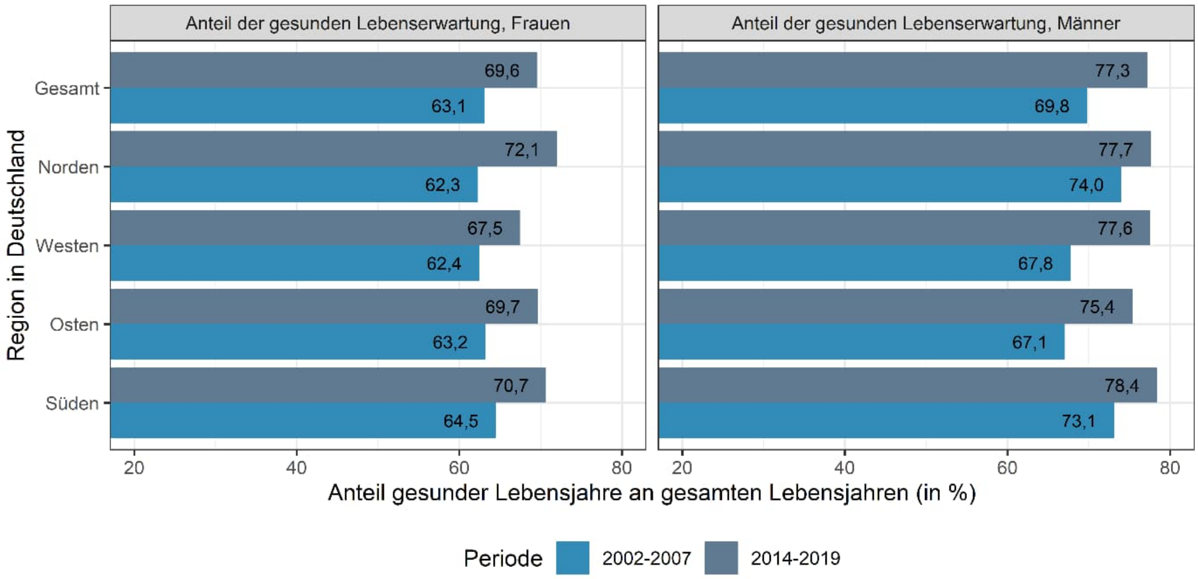

Quelle: Eigene Berechnungen und Abbildung
